# Healthy Kids Out of School: Using Mixed Methods to Develop Principles for Promoting Healthy Eating and Physical Activity in Out-of-School Settings in the United States

**DOI:** 10.5888/pcd11.140207

**Published:** 2014-12-31

**Authors:** Sarah A. Sliwa, Shanti Sharma, William H. Dietz, Peter R. Dolan, Miriam E. Nelson, Molly B. Newman, Maya Rockeymoore, Christina D. Economos

**Affiliations:** Author Affiliations: Shanti Sharma, Peter R. Dolan, Molly B. Newman, Miriam E. Nelson, Christina D. Economos, Tufts University, Boston, Massachusetts; William H. Dietz, Tufts University, Boston, Massachusetts, and George Washington University, Washington, DC; Maya Rockeymoore, Tufts University, Boston, Massachusetts, and Global Policy Solutions, Washington DC.

## Abstract

**Introduction:**

Widespread practices supporting availability of healthful foods, beverages, and physical activity in out-of-school-time (OST) settings would further obesity prevention efforts. The objective of this article was to describe principles to guide policy development in support of healthy eating and physical activity practices in out-of-school settings to promote obesity prevention.

**Methods:**

The Institute of Medicine’s L.E.A.D. framework (Locate Evidence, Evaluate it, Assemble it, and Inform Decisions) was used to identify practices relevant to children’s healthful eating in most OST settings: 1) locate and evaluate information from a national survey of children’s perceptions of healthful-food access; published research, reports, policies and guidelines; and roundtables with OST organizations’ administrators; 2) assemble information to prioritize actionable practices; and 3) inform programmatic direction.

**Results:**

Three evidence-informed guiding principles for short-duration OST resulted: 1) drink right: choose water instead of sugar-sweetened beverages; 2) move more: boost movement and physical activity in all programs; and 3) snack smart: fuel up on fruits and vegetables.

**Conclusion:**

Healthy Kids Out of School was launched to support the dissemination and implementation of these guiding principles in short-duration OST settings, complementing efforts in other OST settings to shift norms around eating and physical activity.

## Introduction

Strategies to accelerate obesity prevention include modifying environments where children spend time in order to increase their opportunities for healthful eating and physical activity (1). Children’s consumption of fruits and vegetables (2) and their levels of physical activity continue to fall short of recommendations (3). Given their broad demographic reach and contact time with children, out-of-school time (OST) programs are well suited to improve children’s access to healthful foods and beverages and increase opportunities for physical activity (4–6). Consistent policies may help (4). 

OST environments include before-school, after-school, and summer programs, which together engage millions of children, including large numbers of minority youths (7) who are at greater risk for obesity (8). These settings vary in their operational structures, leadership, and contact hours with children; they include long-duration after-school programs (which are typically operated 5 days per week for more than 3 hours a day by paid staff) and short-duration programs (which are often led by parents or unpaid volunteers, including youth sports and Scouts).

The Healthy Out-of-School Time Coalition developed voluntary standards for healthful eating and physical activity, which were adopted by the National Afterschool Association (9,10). The standards include 11 overarching guidelines and supporting standards that are well-suited for programs with established training opportunities. However, short-duration OST programs, especially those led by volunteers, have limited training opportunities. These programs would benefit from complementary standards that are easy to understand and implement.

This article describes the process used to identify, develop, and support the implementation of principles to improve children’s energy balance in short-duration OST settings. Mixed methods allowed researchers to integrate quantitative and qualitative data to quantify the reach of OST programs, policies, and guidelines and to engage stakeholders in prioritizing and tailoring a response.

The Tufts University institutional review board reviewed and approved this research.

## Methods

### Using the L.E.A.D. framework to develop overall approach

**Figure 1:**
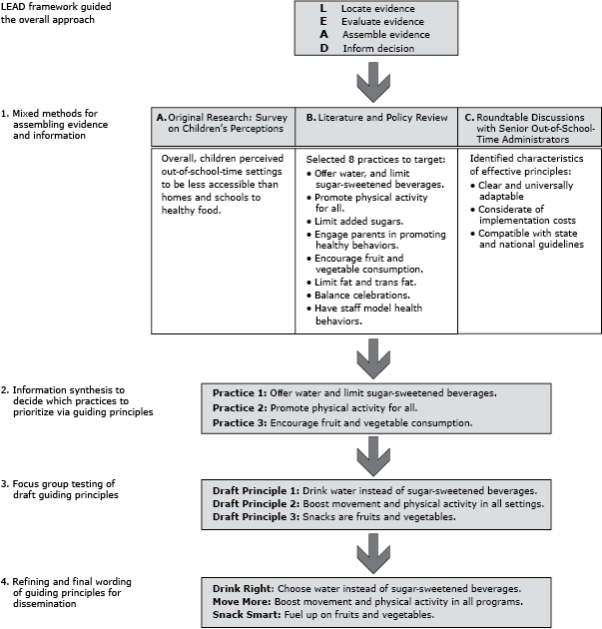
Applying the L.E.A.D. Framework to design a mixed-methods process for developing guiding principles for out-of-school time programs,

We used the Institute of Medicine’s L.E.A.D. framework (11) to develop an explanatory, sequential, mixed-methods process to locate information from a national survey of children’s perceptions of access to healthy foods (original research); published literature, reports, policies, and guidelines on physical activity and nutrition in OST settings; and interviews with senior administrators of national OST organizations. This information was evaluated and assembled to prioritize practices that reflected children’s perceived access to healthful foods, evidence-informed guidelines and recommendations, and OST administrators’ assessments of feasibility. Principles promoting these actions were drafted. Focus groups with local program leaders suggested revisions and implementation needs (Figure 1).

### Mixed methods for assembling evidence and information

Harris Interactive, Rochester, New York, was commissioned to conduct an online survey of the perceptions of children aged 8 to 18 years (N = 1,178) about access to fresh fruits and vegetables, chips, cookies, and candy at home, at school, and in OST settings. The survey was administered in September 2010 with a stratified, nationally representative, random sample of respondents in the United States following methods detailed elsewhere (12). Questions included “How easy or difficult is it for you to get fresh fruits and vegetables at each of the following places?” and “How easy or difficult is it for you to get chips, cookies, or candy at each of the following places?” Respondents assessed the relative ease (very difficult, somewhat difficult, neither easy nor difficult, somewhat easy, very easy, and does not apply) of accessing these foods at home, at school, and at after-school activities. We excluded “does not apply” (<2% fruits and vegetables responses, <1% cookies, chips, and candies responses) and collapsed responses into “very easy” and “not very easy” and compared them using χ^2^ tests with significance set at *P* ≤ .05). Statistical analyses were conducted using SAS 9.2 (SAS Institute Inc.).

Peer-reviewed articles and publicly available white papers were reviewed to identify national OST organizations whose programming collectively reaches large numbers of children of both sexes, including urban and rural youths, specific racial or ethnic groups, and low-income populations. Next, a policy scan provided evidence-informed recommendations and guidelines addressing physical activity, foods, and beverages served in OST settings (Appendix). This scan first collected policies and recommendations from federal programs (eg, the Child and Adult Care Food Program) and national nonprofit and professional entities that use an evidence-informed approach (eg, Institute of Medicine). Lastly, guidelines were gathered from national research institutions and OST organizations. Reference lists were consulted to identify additional sources. To determine whether national OST programs had developed policies, Google and Google Scholar searches were conducted from November 2010 through February 2011 by using the terms policy, guidelines, snack, nutrition, food, wellness, physical activity, and combinations of the terms (using *and* or *or*), with the names of OST organizations identified. A matrix was constructed to compare guidelines and policy language and identify the most frequently targeted practices.

Senior administrators (eg, CEOs) from national OST organizations whose documents we reviewed were engaged to identify practices that could be implemented in various OST settings and to advise on implementation and dissemination needs. Two roundtable meetings were convened in the spring and summer of 2011, with 1 to 2 leaders from 9 organizations: YMCA of the USA, Boys and Girls Clubs of America, Boy Scouts of America, Girl Scouts of the USA, National Council of La Raza, National Urban League, Pop Warner, US Youth Soccer, and National Council of Youth Sports.

### Drafting principles and testing in focus groups

A marketing agency crafted guiding principles to promote the selected evidence-informed practices. Eight focus groups were conducted with local program leaders from 4 OST organizations, Pop Warner, Girl Scouts of the USA, the National Council of La Raza, and the National Urban League. A semistructured interview protocol assessed reactions to and comprehension of the principles (eg, “What does the phrase [keyword] mean to you?”), identified potential revisions, and implementation needs (“What resources would help make this happen?”). Organizations representing scouting, sports, and enrichment programs reaching minority youths were selected to increase researchers’ understanding of diverse OST programs. A point-person in each organization recruited participants via e-mail, phone calls, and fliers. Two focus groups were held per organization, with 5 to 12 participants per group, lasting 45 to 60 minutes each. Focus groups held in Chicago, Illinois; Tampa, Florida; Kansas City, Kansas; and Atlanta, Georgia, engaged 53 participants (35 women, 18 men).

The same trained moderator and note-taker attended all 8 focus groups. Detailed notes and audio recordings were taken at every focus group. Only 4 audio recordings were of adequate quality and were transcribed. After each session, the moderator and note-taker compiled and compared notes and observations.

By using a coding guide based on the interview script, the moderator and an independent reviewer analyzed the transcripts and notes, identified emergent themes, and resolved discrepancies. Statements were analyzed within and across organizations. Broad themes were separated into subthemes as needed, and this iterative process continued until statements appeared under only 1 heading (13). Coders selected representative quotes for each theme.

### Refining and final wording of principles

Focus group findings informed revisions to the principles, which were finalized in consultation with a marketing group (Figure 1).

## Results

Children and adolescents perceived significant differences in “very easy” availability of fresh fruits and vegetables at home, at school, and in OST environments (*P* < .001 for children aged 8 to 12; *P* < .001 for children aged 13 to 18) (Table). Among younger children (aged 8 to 12, n = 509), 26.7% perceived fruits and vegetables to be very easy to access at OST activities. Overall, children aged 8 to 18 perceived very easy access to fruits and vegetables to be lowest in OST settings.

**Table:** Perceptions of Ease of Access to Fresh Fruits and Vegetables in Home, School, and Out-Of-School Settings As Reported by a Nationally Representative Sample of US Children Aged 8 to 18 (N = 1,178)

Age Group	Food Type	Ease of Access	Home (%)	School (%)	Afterschool (%)	*P* Value
**8–12 year olds**	Fruits and vegetables	Very easy	72.2	35.3	26.6	<.001
		All other responses^a^	27.8	64.7	73.4	
	Cookies, chips, and candies	Very easy	30.6	25.0	26.7	.124
		All other responses^a^	69.4	75.0	73.3	
**13–18 year olds**	Fruits and vegetables	Very easy	53.3	20.9	13.8	<.001
		All other responses^a^	46.7	79.1	86.2	
	Cookies, chips, and candies	Very easy	33.9	48.9	37.1	<.001
		All other responses^a^	66.1	51.2	62.9	

^a^ Includes somewhat easy, neither easy nor difficult, somewhat difficult, and very difficult.

Document review identified numerous guidelines for providing healthful snacks and physical activity for long-duration after school programs (ie, programs that operate 5 days a week, and meet for 3 or more hours per day). Few guidelines targeted short-duration OST programs (programs that meet for less than 3 hours per day) and volunteer-led programs (eg, Boy Scouts, youth sports).

Twenty evidence-informed practices for improving the quality of snacks and physical activity in OST settings were identified and further evaluated by nutrition and physical activity experts (C.E., M.N., and W.D.) for their potential impact on energy balance and implementation feasibility. Eight were selected for roundtable discussions:

- Offer water, and limit sugar-sweetened beverages
- Promote physical activity for all
- Limit added sugars
- Engage parents in promoting healthy behaviors
- Encourage fruit and vegetable consumption
- Limit, especially trans fat
- Balance celebrations to feature healthy treats
- Facilitate staff’s role in modeling healthy behaviors

### Roundtable discussions

The roundtable discussions highlighted the diversity of organizational structures in OST settings and the opportunities and barriers to offering physical activity and fruits and vegetables. Senior administrators from each of the 9 programs evaluated the proposed practices, considering relevance to different organizational cultures and structures, compatibility with state and national guidelines, competing priorities, and feasibility.

Roundtable participants emphasized that to be successful, principles needed to be specific and relevant. Policies limiting fat (total and saturated) and especially trans fat were seen as specific but less relevant for sports leagues. Participants selected 3 evidence-informed practices from the 8 and supported developing communications that complemented existing standards and assisting organizations without policies in place. The term “guiding principles” connoted core practices and organizational ethos rather than regulations and was favored over “guidelines,” “policies,” or “standards.” Participants stressed that although they could adopt and disseminate policies organization-wide at the national level, local program leaders determine whether those policies are implemented.

Information assembled through the survey, document review, and roundtables drove the decision to prioritize 3 practices: offer water instead of sugar-sweetened beverages; promote physical activity for all; and serve fruits and vegetables.

Guiding principles were drafted and pilot-tested with local program leaders:

- Draft Principle 1: Drink water instead of sugar-sweetened beverages.
- Draft Principle 2: Boost movement and physical activity in all settings.
- Draft Principle 3: Snacks are fruits and vegetables.

**Draft principle 1: Drink water instead of sugar-sweetened beverages.** Participants in all focus groups identified examples of sugar-sweetened beverages but were unsure whether the term addressed a particular ingredient or all sweetened drinks: “It’s very specific. When you say sugar-sweetened beverages, I think of pure cane sugar-sweetened beverage. I don’t think, oh, it’s sweetened with Splenda or something else.” Participants recommended simpler phrasing: “drink water,” or “drink more water.”

**Draft Principle 2: Boost movement and physical activity in all settings.** “Boost” was interpreted as “do more.” “Movement” and “physical activity” connoted activities of different intensities; however, “all settings” was controversial as it could include locations outside program leaders’ influence or where physical activity would be inappropriate (eg, church): *“*In all settings will be hard because there are some times that physical activity or moving around is not acceptable, you know?”

The most common suggestion was to remove the phrase “in all settings.”

**Draft Principle 3: Snacks are fruits and vegetables:** This principle was seen as bossy, restrictive, and inconsistent with general perceptions of snacks: “Says fruits and vegetables are [the] only good snack —and that’s not true.” The most frequent suggestion was inverting the wording to “Fruits and vegetables are snacks.” Other ideas included “snack on” or “choose” fruits and vegetables.

**Needs assessment for implementation of the principles.** Focus group participants identified resources needed to facilitate principle implementation. For substituting water, these included physical materials (eg, water filters) and educational and promotional materials to build demand for water by making it “cool” or positive. Sports program coaches described pervasive sports drink marketing *—* “Everyone wants G2” *—* and identified promotional water bottles as facilitators and barriers: the bottles are free and easy to fill with water but advertise sports drinks.

To increase physical activity, additional training was requested. Implementation challenges included space limitations, neighborhood safety, staff capacity, liability concerns, and equipment costs. Leaders from programs offering homework assistance and with parent volunteers anticipated time constraints: “We volunteer. We work. We have kids — not a lot of time to figure it out. . . . If you want this to happen you have to make it accessible. Take all the guess-work out.”

Barriers to serving fruits and vegetables included time, preparation space, storage, and perishability. Sports program coaches observed that sugar-sweetened beverages, candy, and salty snacks were widely available at concession stands and conflict with snacking healthfully on game days. Partnerships with food distributors, supermarkets, and community organizations were identified as desirable cost-containment strategies. Participants also requested peer-to-peer learning opportunities.

**Figure 2:**
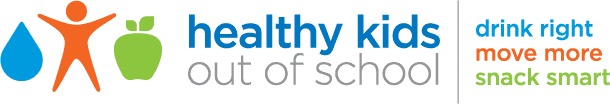
Healthy Kids Out of School promotional logo and taglines.

**Final outcome: 3 guiding principles.** The principles were reworded, with input from a marketing agency, to incorporate focus group feedback. The term “sugar-sweetened beverage” was retained; emphasis on substitution was central to the principle’s potential to influence energy balance. Materials promoting this principle simplified this message graphically (eg, a water droplet with the tagline “Drink Right”). The full principles and rationale are described in supporting materials (Figure 2):

- Drink right: Choose water instead of sugar-sweetened beverages.
- Move more: Boost movement and physical activity in all programs.
- Snack smart: Fuel up on fruits and vegetables.

## Discussion

The broad objective of our study was to make healthy eating and physical activity more accessible to all children in OST settings. The L.E.A.D framework informed an explanatory sequential mixed-methods process for identifying and prioritizing healthy practices relevant to diverse OST programs. The gaps identified in the policy scan informed the decision to launch Healthy Kids Out of School (HKOS) with a focus on short-duration OST programs. The implementation of principles to displace sugar-sweetened beverages with water, increase physical activity, and promote fruits and vegetables promises to provide healthy options for children in OST.

Input at national and local levels was essential to gauging feasibility. Engaging national OST administrators ensured that principles reflected organizational priorities. Local program leaders identified implementation challenges consistent with previous research (14,15). The successful implementation of similar strategies demonstrates their feasibility (4,16–20). Children’s perceptions were also essential because children recognize the contribution of unhealthful foods to obesity, associate obesity with adverse outcomes (12), and may be receptive to healthy options. Although nutrition experts collaborated with professional survey developers to develop the questions, the lack of a priori validity and reliability testing is a limitation of our study.

To disseminate the guiding principles and support their adoption, the HKOS initiative was launched in November 2011 with 11 partner OST organizations, including those involved with the focus groups. Collectively, these organizations reach tens of millions of children and adolescents (7,21–23). Post-launch, the HKOS-Healthy Kids Hub website (http://www.healthykidshub.org/) was developed to support nationwide dissemination and connect program leaders to resources. Program-specific goals for healthy snacks, beverages, and physical activity in OST settings communicate implementation targets (24). HKOS is an initiative of ChildObesity180 at Tufts University. With leadership from public, private, academic, and nonprofit sectors, ChildObesity180 implements integrated strategies to reverse childhood obesity trends.

Implementation efforts in 3 New England states seek to engage OST program leaders, identify customizations that help integrate the principles within each organization’s context and culture, and disseminate the principles within existing policy and communication channels. Efforts are being evaluated in a 3-year study using the Reach Effectiveness Adoption Implementation Maintenance (RE-AIM) framework (25). The mixed-methods evaluation includes a validated survey assessing snacks and beverages and opportunities for physical activity at program meetings and observations and key informant interviews conducted during site visits. This regional effort will inform HKOS efforts nationwide. A national evaluation is needed to assess the feasibility and effectiveness of large-scale, policy-based approaches to enhancing OST settings for obesity prevention.

By complementing existing recommendations, policies, and programs, HKOS guiding principles may initiate novel changes in short-duration OST programs. Many children participate in OST programs, even multiple programs, throughout the year. Providing consistent access to healthful foods and physical activity will help shift behavioral norms.

## Appendix. Recommendations, Guidelines, and Evaluation Tools Consulted in Policy Review

This file is available for download as a Microsoft Word document.

## References

[1] Glickman DL , Sim LJ , Del Valle Cook H , Ann Miller E , editors. Accelerating progress in obesity prevention: solving the weight of the nation. Washington (DC): The National Academies Press; 2012. 1-16,154, 442. 24830053

[2] Kim SA , Moore LV , Galuska D , Wright AP , Harris D , Grummer-Strawn LM , Vital signs: fruit and vegetable intake among children — United States, 2003–2010. MMWR Morb Mortal Wkly Rep 2014;63(31):671–6. 25102415 PMC4584658

[3] Gortmaker SL , Lee R , Cradock AL , Sobol AM , Duncan DT , Wang YC . Disparities in youth physical activity in the United States: 2003–2006. Med Sci Sports Exerc 2012;44(5):888–93. 10.1249/MSS.0b013e31823fb254 22089478

[4] Beets MW , Tilley F , Kim Y , Webster C . Nutritional policies and standards for snacks served in after-school programmes: a review. Public Health Nutr 2011;14(10):1882–90. 10.1017/S1368980011001145 21729480

[5] Beets MW , Beighle A , Erwin HE , Huberty JL . After-school program impact on physical activity and fitness: a meta-analysis. Am J Prev Med 2009;36(6):527–37. 10.1016/j.amepre.2009.01.033 19362799

[6] National Physical Activity Plan. National physical activity plan for the United States; 2010. http://www.physicalactivityplan.org/NationalPhysicalActivityPlan.pdf. Accessed November 1, 2010.

[7] Afterschool Alliance, JC Penney Afterschool. America after 3pm: the most in-depth study of how America’s children spend their afternoons ; 2009 . http://www.afterschoolalliance.org/documents/AA3PM_National_2009.pdf. Accessed November 1, 2010 .

[8] Singh GK , Kogan MD , van Dyck PC . A multilevel analysis of state and regional disparities in childhood and adolescent obesity in the United States. J Community Health 2008;33(2):90–102. 10.1007/s10900-007-9071-7 18049885

[9] Wiecha JL , Hall G , Gannett E , Roth B . Development of healthy eating and physical activity quality standards for out-of-school time programs. Child Obes 2012;8(6):572–6. 23181923 10.1089/chi.2012.0030

[10] Wiecha J , Hall G , Roth B . National Afterschool Association standards for healthy eating and physical activity in out-of-school time programs; 2011. Accessed December 13, 2013. http://www.niost.org/pdf/host/Healthy_Eating_and_Physical_Activity_Standards.pdf

[11] Kumanyika K , Parker L , Sim LS , editors. Bridging the evidence gap in obesity prevention: a framework to inform decision making. Washington (DC): The National Academies Press; 2010. 336p. 25032376

[12] Economos CD , Bakun PJ , Herzog JB , Dolan PR , Lynskey VM , Markow D , Children's perceptions of weight, obesity, nutrition, physical activity and related health and socio-behavioural factors. Public Health Nutr 2014;17(1):170–8. 10.1017/S136898001200479X 23199642 PMC10282499

[13] Krueger RA , Casey MA , Kumar A . Focus groups: a practical guide for applied research. Thousand Oaks (CA): Sage Publications; 2008. p.113-128.

[14] Mozaffarian RS , Andry A , Lee RM , Wiecha JL , Gortmaker SL . Price and healthfulness of snacks in 32 YMCA after-school programs in 4 US metropolitan areas, 2006-2008. Prev Chronic Dis 2012;9:E38. 22239753 10.5888/pcd9.110097 PMC3310067

[15] Hastmann TJ , Bopp M , Fallon EA , Rosenkranz RR , Dzewaltowski DA . Factors influencing the implementation of organized physical activity and fruit and vegetable snacks in the HOP'N After-School Obesity Prevention Program. J Nutr Educ Behav 2013;45(1):60–8. 10.1016/j.jneb.2012.06.005 23178043

[16] Mozaffarian RS , Wiecha JL , Roth BA , Nelson TF , Lee RM , Gortmaker SL . Impact of an organizational intervention designed to improve snack and beverage quality in YMCA after-school programs. Am J Public Health 2010;100(5):925–32. 10.2105/AJPH.2008.158907 19833987 PMC2853616

[17] Gortmaker SL , Lee RM , Mozafarrian RS , Sobol AM , Nelson TF , Roth BA , Effect of an after-school intervention on increases in children’s physical activity. Med Sci Sports Exerc 2012;44(3):450–7. 10.1249/MSS.0b013e3182300128 21814151

[18] Rosenkranz RR , Behrens TK , Dzewaltowski DA . A group-randomized controlled trial for health promotion in Girl Scouts: Healthier Troops in a SNAP (Scouting Nutrition & Activity Program). BMC Public Health 2010;10(81):1–13. 20170502 10.1186/1471-2458-10-81 PMC2832775

[19] Jago R , Baranowski T , Baranowski JC , Thompson D , Cullen KW , Watson K , Fit for Life Boy Scout badge: outcome evaluation of a troop and Internet intervention. Prev Med 2006;42(3):181–7. 10.1016/j.ypmed.2005.12.010 16458955

[20] Thompson D , Baronowski T , Baranowski J , Cullen K , Jago R , Watson K , Boy Scout 5-a-Day Badge: outcome results of a troop and Internet intervention. Prev Med 2009;49(6):518–26. 10.1016/j.ypmed.2009.09.010 19765608

[21] Report on trends and participation in organized youth sports: market research report. Stuart (FL): National Council on Youth Sports; 2008. http://www.ncys.org/pdfs/2008/2008-ncys-market-research-report.pdf. Accessed Decem ber 11, 2014.

[22] Overview of Boy Scouts of America. Boys Scouts of America; 2013. http://www.scouting.org/About/FactSheets/OverviewofBSA.aspx. Accessed December 13, 2013.

[23] About Girl Scouts of the USA. Girl Scouts of America; 2013. http://www.girlscouts.org/who_we_are/facts/pdf/facts_gs.pdf. Accessed December 13, 2013.

[24] ChildObesity180 nutrition and physical activity goals. Boston (MA): ChildObesity180 ; 2013. http://www.childobesity180.org/sites/default/files/documents/ChildObesity180%20Nutrition%20and%20Physical%20Activity%20Goals.pdf#sthash.9vFQHduE.dpuf. Accessed March 27, 2014.

[25] Glasgow RE , Vogt TM , Boles SM . Evaluating the public health impact of health promotion interventions: the RE-AIM framework. Am J Public Health 1999;89(9):1322–7. 10.2105/AJPH.89.9.1322 10474547 PMC1508772

