# Supplementary material for: Healthy Kids Out of School: Using Mixed Methods to Develop Principles for Promoting Healthy Eating and Physical Activity in Out-of-School Settings in the United States
Source: Prev Chronic Dis. 2014 Dec 31;11:E227. doi: 10.5888/pcd11.140207 (PMC4283424; doi:10.5888/pcd11.140207)
Supplement: Supplementary file 1 [file 14_0207_01.docx]

**Appendix**

**Recommendations, Guidelines, and Evaluation Tools Consulted in Policy Review**

**National Recommendations and Guidelines**

Food and Nutrition Service (2010). Afterschool Snacks in the National School Lunch Program. Available at: http://www.fns.usda.gov/cnd/afterschool/factsheet.htm

Food and Nutrition Service (2010). Child and Adult Care Feeding Program. Available at: http://www.fns.usda.gov/cnd/care/cacfp/aboutcacfp.htm

Healthy Out-Of-School Time Coalition. (2011) *Recommended National Standards for Healthy Eating and Physical Activity in Out-of-School Time Programs*. Available at: http://www.niost.org/pdf/host/Healthy_Eating_and_Physical_Activity_Standards.pdf

Institute of Medicine. (2010) *Child and Adult Care Food Program: Aligning Dietary Guidance for All. Washington, D.C.* The National Academies Press.

Institute of Medicine. (2007). *Nutrition Standards For Foods in Schools: Leading the Way Toward Healthier Youth.* Washington, D.C.: The National Academies Press.

National Physical Activity Plan Alliance. The National Physical Activity Plan (2010) at: http://www.physicalactivityplan.org

**State, Community, and Program Level Recommendations and Guidelines**

Action for Healthy Kids. Local School Wellness Policies: Classroom Celebrations and Parties.http://www.schoolnutrition.org/Content.aspx?id=7428

California Department of Education Nutrition Services Division. (2008) *Nutrition Standards for Snacks In After School Programs*. Available at: www.cde.ca.gov/ls/nu/as/documents/**afterschool**nutstan.doc and http://www.cde.ca.gov/ls/nu/as/afterschoolnutstan.asp

Harvard Prevention Research Center.(2010) *Harvard Prevention Research Center Environmental Standards for Nutrition and Physical Activity in Out-of-School Time Programs*. *Food and Fun After-School: 2nd Edition.* Available at: http://www.hsph.harvard.edu/research/prc/files/nutrition_and_pa_environmental_standards_for_ost_061710.pdf

Harvard Prevention Research Center. (2010)*Family Engagement Planning Tool*. *Food and Fun After-School: 2nd Edition.* Available at: www.hsph.harvard.edu/.../family_engagement_planning_tool_7.2010.doc

Commonwealth of Massachusetts Department of Public Health. (2011) *Regulations for Nutrition Standards for Competitive Foods in Schools.* Available at: http://www.mahealthcouncil.org/2011-02-09_DPH_Nutrition_Standards.pdf

**Assessment Tools/Criteria**

Alliance For A Healthier Generation. *Before and Afterschool Toolkit.* http://www.healthiergeneration.org/uploadedFiles/For_Schools/_New_Builder_Pages/Toolkits/BeforeAfterschoolToolkit.pdf

Boston Medical Center. *After School Physical Activity and Nutrition Self-Assessment Tool* (AS-PANSAT). Available at: http://supportunitedway.org/images/chapters/ch8.pdf

# California AfterSchool Network. California After School Program Quality Self-Assessment Tool (QSA). http://www.afterschoolnetwork.org/post/california-after-school-program-quality-self-assessment-tool

Food Research and Action Center. *The Afterschool Nutrition Standards of Excellence. At: http://www.afterschoolresources.org/kernel/images/FRAC-%20afstandards%5B1%5D.pdf*

University of North Carolina, North Carolina Department of Health and Human Services, North Carolina Prevention Partners. (2003) *Nutrition and Physical Activity Self-Assessment for Child-Care (NAP SACC*) at: https://riskfactor.cancer.gov/mfe/instruments/benjamin-nap-sacc-nutrition-and-physical-activity-self-assessment-for-child-care

YMCA Activate America. (2008). *Community healthy living index-- afterschool child care site.* at http://www.ymca.net/communityhealthylivingindex/tools.html
